# Novel compound heterozygous variants in the *RPL3L* gene causing dilated cardiomyopathy type-2D: a case report and literature review

**DOI:** 10.1186/s12920-023-01567-y

**Published:** 2023-06-12

**Authors:** Qi Yang, Qiang Zhang, Zailong Qin, Shujie Zhang, Sheng Yi, Shang Yi, Qinle Zhang, Jingsi Luo

**Affiliations:** 1 Guangxi Key Laboratory of Birth Defects Research and Prevention, Guangxi Key Laboratory of Reproductive Health and Birth Defects Prevention, Maternal and Child Health Hospital of Guangxi Zhuang Autonomous Region, No. 59, Xiangzhu Road, Nanning, China; 2 Department of Genetic and Metabolic Central Laboratory, Maternal and Child Health Hospital of Guangxi Zhuang Autonomous Region, Nanning, China

**Keywords:** Dilated cardiomyopathy type-2D, *RPL3L*, Whole exome sequencing, Novel variants

## Abstract

**Background:**

Dilated cardiomyopathy type-2D (CMD2D) is a rare heart disease causing a severe cardiomyopathy with neonatal onset and rapid progression to cardiac decompensation and death in untreated patients. CMD2D is an autosomal recessive disease resulting from variants in the *RPL3L* gene, which encodes the 60 S ribosomal protein exclusively expressed in skeletal and cardiac muscle and plays an essential role in myoblast growth and fusion. Previous reports have only associated CMD2D with a small duplication and seven nucleotide substitution in the *RPL3L* gene.

**Case presentation:**

In this study, we report the case of a 31 days old Chinese infant patient with severe dilated cardiomyopathy (DCM) and rapid decompensation along with other cardiac malformations. In addition to previously reported clinical features, the patient showed the previously unreported complication of occasional premature atrial contractions and a first-degree atrioventricular block. Whole-exome sequencing (WES) revealed compound heterozygous variants (c.80G > A (p.Gly27Asp) and c.1074dupA (p.Ala359fs*6)) in RPL3L (NM_005061.3). The latter novel variant may result in the absence of protein production with a significant decrease in mRNA level, suggesting it is a loss-of-function mutation.

**Conclusions:**

This is the first case report of *RPL3L*-associated neonatal dilated cardiomyopathy in China. The molecular confirmation of the patient expands the genetic spectrum of CMD2D, and the clinical manifestation of CMD2D in the patient provides additional clinical information regarding this disease.

## Introduction

Dilated cardiomyopathy (DCM) type 2D (CMD2D, MIM 619,371) is a rare autosomal recessive disorder characterized by the neonatal onset of severe cardiomyopathy with rapid progression to cardiac decompensation and death unless the patient undergoes heart transplantation [1]. It is caused by homozygous or compound heterozygous mutations in the ribosomal protein large 3-like (*RPL3L*, NM_005061, MIM 617,416) gene, located at 16p3.3, which contains 10 exons and encodes the 60 S ribosomal protein [2]. It is exclusively expressed in skeletal and cardiac muscle and is essential in myoblast growth and fusion [3–5]. To date, only eight mutations have been described in the *RPL3L* gene associated with CMD2D [1, 6, 7]. Almost all identified variants are missense variants, which are predicted to disrupt the binding of RPL3L to the 60 S ribosomal subunit. Additional reports on these gene variants and their phenotypes will be critical to the understanding of this condition. Herein, we report compound heterozygous *RPL3L* variants, c.80G > A (p.Gly27Asp) and c.1074dupA (p.Ala359fs*6), identified in a Chinese patient with severe DCM .

## Case presentation

**Fig. 1:**
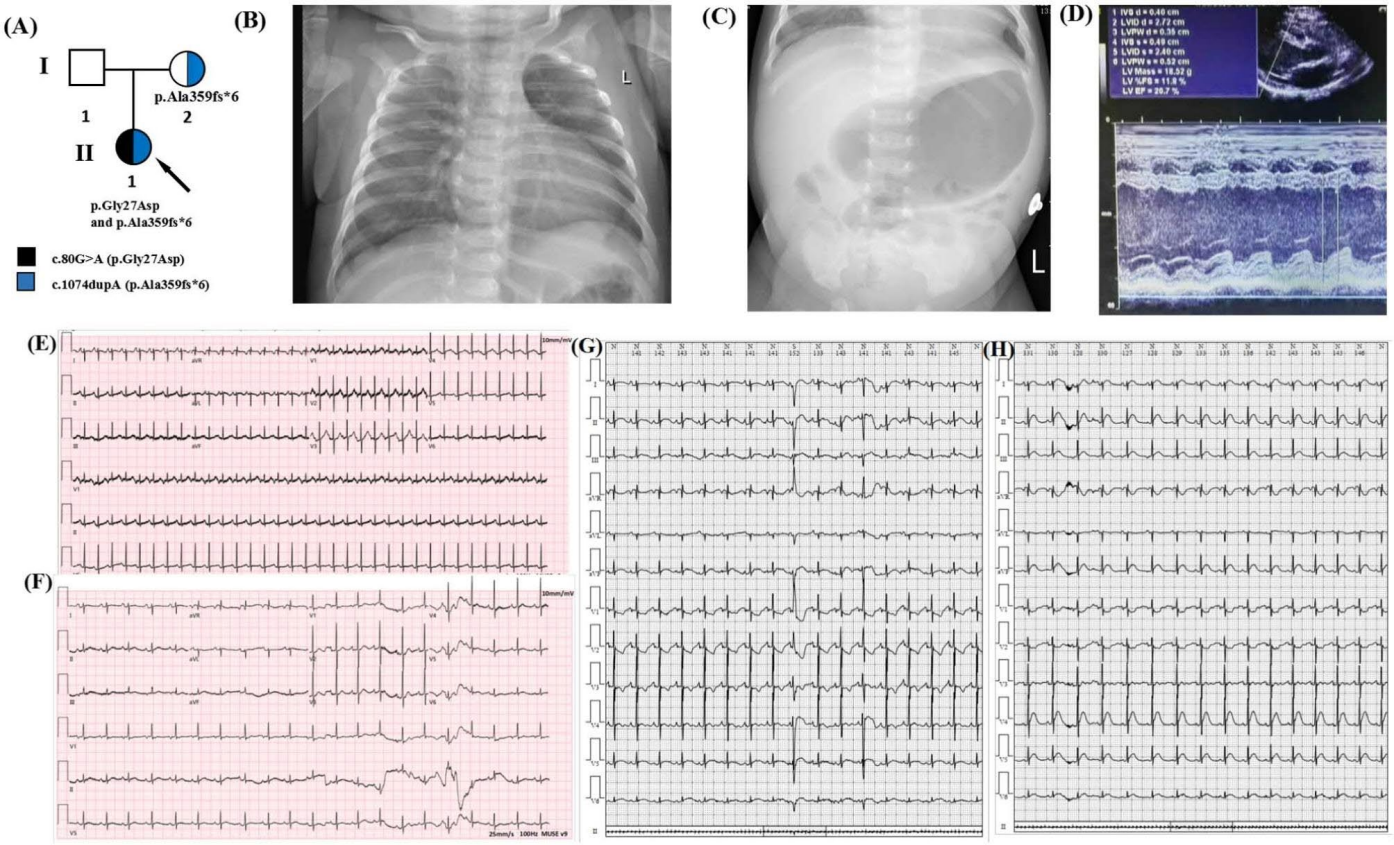
**(A)** Pedigree chart of the family of the patient with dilated cardiomyopathy type-2D. The proband is indicated by a black arrow. **(B)-(C)** Chest and abdominal X-ray showing severe cardiomegaly and intestinal obstruction at presentation. **(D)** M-mode echocardiography displaying depressed LV systolic function (LVSF 12%). **(E)-(F)** Electrocardiogram showing T segmentchanges and first degree atrioventricular block. **(G)-(H)** 24-hour EKG monitoring showing occasional premature atrial contractions and paroxysmal ST segment changes

The proband (II:1) was the femal infant and the first child of healthy non-consanguineous Chinese parents (Fig. 1A). She was born by normal vaginal delivery at 39 weeks and had normal cry at birth; her birth weight was 3.1 kg, length was 49.9 cm, head circumference was 34.4 cm, and APGAR scores at 1 and 5 min were 9 and 10, respectively. At 31 days old, she was admitted to the hospital for sudden-onset irritability, respiratory distress, hypoxia, and being cold. The patient was transferred immediately to the NICU, where she was given continuous positive airway pressure therapy. The patient was initially diagnosed with Sepsis-Induced Cardiomyopathy. Physical examination showed that her heart rate was 206 bpm, her respiratory rate was 54 bpm, and she had a protuberant abdomen, mild hypotension, and respiratory distress. The initial laboratory results showed severe metabolic acidosis (pH = 7.05, pCO_2_ = 14 mmHg, PO_2_ = 424mmHg, HCO_3_ = 3.9 mmol/L, anion gap = 16.3 mmol/L, elevated lactate > 16.8 mmol/L, hyperammonemia (175.0 µmol/l), elevated NT brain-type pro-natriuretic peptide (NTpro-BNP) > 35,000 pg/mL, elevated creatinine (90 µmol/L), and elevated liver enzymes (AST 169U/L and ALT 105 U/L). The chest radiograph showed a thickened texture in both lungs with patchy faint shadows and enlarged heart shadows (Fig. 1B). An abdominal X-ray showed an abnormal intestinal gas shadow and partial intestinal wall thickening, suggesting intestinal obstruction (Fig. 1C). An echocardiogram showed a severe dilated left ventricle, severe decreased LV shortening fraction (FS) (12%), patent foramen ovale, and mild tricuspid and mitral valve regurgitation (Fig. 1D). Her EKG monitoring showed a sinus rhythm with an average heart rate of 137 bpm, a first-degree atrioventricular block, occasional premature atrial contractions, and paroxysmal ST and T segment changes (Fig. 1E–H). Upon admission to Guangxi Maternal and Child Health Hospital on day 2, infection testing was negative for respiratory pathogens including influenza, common respiratory pathogens, influenza viruses A and B, parainfluenza virus 1–4, respiratory syncytial virus, rhinovirus, metapneumovirus, mycoplasma pneumonia, and chlamydia pneumoniae. Serologies for common causes of viral myocarditis, including COVID-19, Epstein-Barr Virus, adenovirus, coxsackievirus, Herpes virus 1–2, parvovirus B19, enterovirus, and HIV were also negative. Based on the clinical symptoms and biochemical indicators, a plausible diagnosis of hereditary dilated cardiomyopathy was made. She was treated with milrinone at a dose of 0.5 µg/kg/min. After combination therapy for one month, she was discharged from the hospital with oral digoxin, furosemide, spironolactone, captopril, prednisone and coenzyme Q10. She is now 5 months old and in a stable condition.

**Fig. 2:**
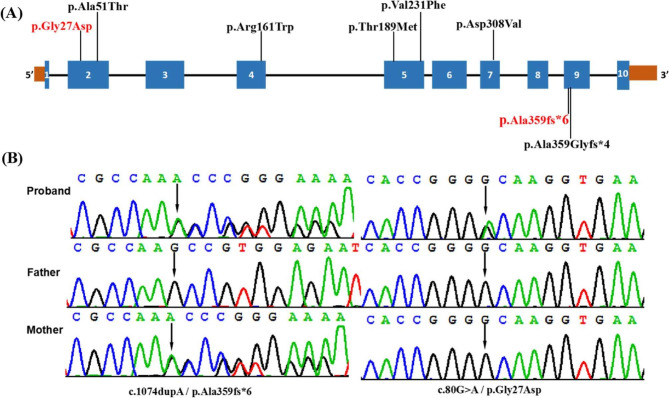
**(A)** The distribution of all variants detected so far in eight dilated cardiomyopathy patients with RPL3L variants. **(B)** Sanger sequencing DNA chromatograms of RPL3L indicating the frameshift c.1074dupA(p.Ala359fs*6) variant inherited from the mother and the missense variant c.80G > A / p.Gly27Asp was a *de novo* variant

WES was applied to identify the potential gene mutation of the proband with DCM. WES generated approximately 5.5 Gb of data with 99.6% coverage of targeted regions, and 97.7% of targets were sequenced > 20 times. Genetic analysis revealed two heterozygous variants in *RPL3L* gene: c.80G > A (p.Gly27Asp) and c.1074dupA (p.Ala359fs*6) (Fig. 2A-B). These variants were verified by PCR and Sanger sequencing in proband and her parents. Sanger sequencing further revealed that the heterozygous c.1074dupA (p.Ala359fs*6) variant was identified in her mother, while the heterozygous c.80G > A (p.Gly27Asp) was absent in both parents. Further paternity was confirmed by microsatellite typing, and the c.80G > A (p.Gly27Asp) variant was proved to be de *novo*.

## Discussion

CMD2D is a rare inherited structural heart disease. In 2020, Mythily et al. reported that *RPL3L* mutations were found in five patients with DCM from three unrelated families through WES. Since then, eight patients with pathogenic mutations in this gene have been described [1, 6, 7]. In the present study, we performed WES and identified compound heterozygous variants in the *RPL3L* gene in a female Chinese infant with severe DCM. Our patient showed common phenotypes associated with CMD2D, including severe DCM, patent foramen ovale, mild tricuspid and mitral valve regurgitation, first-degree atrioventricular block, occasional premature atrial contractions, and paroxysmal ST and T segment changes. Notably, the premature atrial beats and atrioventricular block in the patient we describe have not been previously reported in patients with *RPL3L* pathogenic variants. This is the first report of arrhythmia and first-degree atrioventricular block caused by variants in the *RPL3L* gene.

To date, eight *RPL3L* gene mutations have been identified in eight CMD2D patients [1, 6, 7] (Fig. 2A). Except for one frameshift variant, all reported variants were missense variants. In the patient described in the current study, the biallelic *RPL3L* variants identified were a combination of a frameshift and missense variant. c.1074dupA(p.Ala359fs*6) is a novel variant that is not present in the Human Gene Mutation Database (http://www.hgmd.cf.ac.uk/ac/), HPSD (http://liweilab.genetics.ac.cn/HPSD/), and dbSNP (http://www.ncbi.nlm.nih.gov/SNP/), nor is it present in ExAC or gnomAD (https://gnomad.broadinstitute.org/). It is located in the ninth exon of the *RPL3L* gene and causes a frameshift alteration after codon 359 (Ala), leading to a premature termination codon located at codon 6. The p.Ala359fs*6 variant may act similarly to other previously reported loss-of-function variants (including frameshifts, nonsense variants, and splice sites) of *RPL3L*, like p.Ala359Glyfs*4. These variants are likely to result in the absence of protein production with a significant decrease in mRNA level, which would be degraded via nonsense-mediated mRNA decay [7]. The other variant, c.80G > A (p.Gly27Asp), is a *de novo* variant previously reported in affected individuals [1, 5]. The mutation is located near the ribosomal RNA and negatively charged aspartic acid causes electrostatic repulsion, weakening the binding of RPL3L to the ribosomal RNA [1]. Five patients with DCM from three unrelated families in Spain, the USA, and China have been found to carry the same variant, c.80G > A (p.Gly27Asp), in *RPL3L*, demonstrating a mutational hotspot of *RPL3L*. According to the ACMG/AMP standards and guidelines [8], the novel c.1074dupA (p.Ala359fs*6) variant is pathogenic based on the PVS1, PM2_supporting, and PM3 criteria, while the c.80G > A (p.Gly27Asp) variant is pathogenic according to the PS2, PM1, PM2_supporting, PM3_Strong, and PP3 criteria.

To analyze the genotype and phenotype correlations of RPL3L mutations, we summarized all mutations in *RPL3L* and the clinical phenotype of all cases (including our case) in Table 1 [1, 6, 7]. Some common phenotypes were observed. All patients presented with severe DCM in the neonatal period and rapidly progressed to cardiac decompensation and death without cardiac transplantation. Mitral regurgitation (75%), tricuspid regurgitation (50%), and ST-T abnormalities (75%) are also common. Although we observed convergence phenotypes, we also noticed some variable cardiac malformations, including ventricular septal defect (one patient), patent foramen ovale (two patients), atrioventricular block (one patient), right bundle branch block (one patient), and pulmonary hypertension (one patient). Even patients with the same mutation type can have different phenotypes. For instance, arrhythmia and primary atrioventricular block were only observed in our patients with frameshift mutation p.Ala359Glyfs*4. The underlying mechanism of RPL3L causing DCM remains to be elucidated. RPL3L is exclusively expressed in skeletal muscle and heart tissue and is dynamically regulated in response to external stimuli [2–5]. RPL3L plays an essential role in myoblast growth and fusion [5]. Previous studies have shown that RPL3L variants destabilize the 60 S subunit and affect ribosomal translation, leading to neonatal DCM [1]. The missense variant p.Ala75Val and the splice-donor variant c.1167 + 1G > A have also been reported to be associated with an increased risk of atrial fibrillation [9]. RPL3L gene mutations cause atrial fibrillation, probably because it prolongs P-wave duration and alters neoatrial conduction [10]. Moreover, another variant of the RPL3L gene, c.724 C > T (p.R242W), has been reported in a 6-year-old child with catecholaminergic polymorphic ventricular tachycardia [11]. These findings suggest that RPL3L affects the function of cardiomyocytes and the electrical activity of the heart. The diverse phenotypes observed also indicates that variants occurring at different positions may have site-specific effects on phenotypes. Functional studies of these variants are needed to enhance our understanding of the disease and its mechanisms of action.

**Table 1:** Summary of the clinical features of the patients

Clinical features	Our patient	Ganapathi et al.(2020)					Hemanth et al.(2021)		Bibhuti et al(2022).
	Patient 1	Patient 2	Patient 3	Patient 4	Patient 5	Patient 6	Patient 7	Patient 8	Patient 9
Variants in RPL3L (NM_005061)	c.80G > A (p.Gly27Asp) and c.1074dupA (p.Ala359fs*6)	c.923 A > T (p.Asp308Val) and c.1027 C > T (p.Arg343Trp)	c.923 A > T (p.Asp308Val) and c.1027 C > T (p.Arg343Trp)	c.566 C > T (p.Thr189Met) and c.922G > A (p.Asp308Asn)	c.80G > A (p.Gly27Asp) and c.481 C > T (p.Arg161Trp)	c.80G > A (p.Gly27Asp) and c.481 C > T (p.Arg161Trp)	c.1076_1080delCCGTG (p.Ala359Glyfs*4) and c.80G > A (p.Gly27Asp)	c.1076_1080delCCGTG (p.Ala359Glyfs*4) and c.80G > A (p.Gly27Asp)	c.151 G > A (p.Ala51Thr) and c.691G > T (p.Val231Phe)
Gender	Female	Female	Male	Male	Female	Male	Male	Male	Female
Age at Diagnosis Of DCM	Day31	Day1	Day6	Day75	Day48	Day12	Day1	Day54	Day60
Country	China	Germany	Germany	Colombia	Spain	Spain	USA	USA	USA
Additional Cardiac Findings	PFO, PAC, TR, AB, MR and ST abnormalities,	PFO and PH	TR	TR, MR RBBB ST-T abnormalities	MR ST and T abnormalities	MR, TR. ST and T abnormalities, VSD	MR ST and T abnormalities	MR ST and T abnormalities	NA
Alive/Dead	Alive at 3 months	Died 21st DOL	Died 15th DOL	HT at 6 months Alive at 9 years	HT at 5 months Alive at 10 years	Died 30th DOL	Died 124th DOL	Died 56th DOL	HT at 2 months Alive at 5 months

The following abbreviations are used: DCM: dilated cardiomyopathy; PFO: patent foramen ovale; PAC: Premature atrial contractions, TR: tricuspid regurgitation; MR: mitral regurgitation; AB: atrioventricular block; ST: a segment in ECG from the beginning of S wave until the beginning of T wave; PH = pulmonary hypertension; RBBB: right bundle branch block; VSD:Ventricular septal defect; DOL: day of life; HT: heart transplantation

## Conclusions

In summary, we uncovered a compound heterozygous variant in the *RPL3L* gene in a female Chinese infant with serve DCM with rapid decompensation. This is the first description of a non-consanguineous Chinese pedigree with *RPL3L* variants. These variants associated with severe neonatal DCM, patent foramen ovale, tricuspid and mitral valve regurgitation, first-degree atrioventricular block, occasional premature atrial contractions, and paroxysmal ST segment changes, which further extends the phenotype spectrum of *RPL3L* variations.

## Data Availability

The datasets presented in this study can be found in online repositories. The names of the repository/repositories and accession number(s) can be found at: https://www.ncbi.nlm.nih.gov/sra/PRJNA940950.

## Abbreviations

CMD2D: Dilated cardiomyopathy type-2D
DCM: Dilated cardiomyopathy
WES: Whole-exome sequencing
ACMG: American College of Medical Genetics
AMP: Association of Molecular Pathology guidelines
